# Building a better blood-brain barrier

**DOI:** 10.7554/eLife.31808

**Published:** 2017-10-10

**Authors:** Courtney Lane-Donovan, Joachim Herz

**Affiliations:** 1 Department of NeurologyUniversity of California San FranciscoSan FrancisoUnited States; 2 Department of Molecular GeneticsUniversity of Texas Southwestern Medical CenterDallasUnited States; 3 Department of NeuroscienceUniversity of Texas Southwestern Medical CenterDallasUnited States; 4 Department of Center for Translational Neurodegeneration ResearchUniversity of Texas Southwestern Medical CenterDallasUnited States; 5 Department of Neurology and NeurotherapeuticsUniversity of Texas Southwestern Medical CenterDallasUnited States

**Keywords:** Alzheimer disease, tissue engineering, lipoprotein, cerebrovasculature, Human

## Abstract

A new three-dimensional model of the blood-brain barrier can be used to study processes that are involved in neurodegenerative diseases.

**Related research article** Robert J, Button EB, Yuen B, Gilmour M, Kang K, Bahrabadi A, Stukas S, Zhao W, Kulic I, Wellington CL. 2017. Clearance of beta-amyloid is facilitated by apolipoprotein E and circulating high-density lipoproteins in bioengineered human vessels. *eLife* **6**:e29595. doi: 10.7554/eLife.29595

Over 100 years have passed since the German psychiatrist Aloysius Alzheimer first described the ravages of the disease that now bears his name. It took 80 years to unravel the first clues to the genetic and molecular basis of Alzheimer’s disease, which is the most common form of age-related dementia. And although we now have a more detailed picture of what causes the accumulation of amyloid plaques and neurofibrillary tangles that are still used to diagnose the disease, we still lack effective treatments.

In the early 1990s, researchers discovered that an isoform of apolipoprotein E – a protein whose primary role is to mediate the transport of lipids in the blood – increases the risk of developing Alzheimer’s three-fold in people with one copy of the gene for this isoform and twelve-fold in people with two copies of the gene (Corder et al., 1993). We now know that apolipoprotein E (ApoE) has many functions in the brain, including the clearance of beta-amyloid peptides (which are aggregates of proteins that can stick together to form fibrils) from the brain. However, the different isoforms affect the clearance to different degrees: the isoform ApoE4 severely impedes clearance, ApoE3 has a moderate effect, and ApoE2 has a minimal effect (Castellano et al., 2011). However, we do not understand how ApoE4 instigates neurodegeneration or the mechanisms by which the different isoforms affect amyloid clearance.

In the brain, a structure called the blood-brain barrier controls the movement of metabolites, toxins and cells from the brain to the blood. If this structure is disrupted, the resulting build-up of toxins has been implicated in many neurological disorders, including Alzheimer’s (Sagare et al., 2012). In the past it has been difficult to study the blood-brain barrier because there are too many variables to control for in animal models, while 2D cell-culture models are unable to mimic the unique features of blood vessels found in the brain. Now, in eLife, Cheryl Wellington of the University of British Columbia and colleagues – including Jérôme Robert as first author – report a new 3D model of the blood-brain barrier that can be used to study how ApoE removes amyloid (Robert et al., 2017).

Blood vessels consist of two key cell types: multiple layers of smooth muscle cells that provide tone and structure, and endothelial cells that cover the muscle cells to form a protective barrier. For over a decade, researchers have used an in vitro model called a bioreactor that is made by growing these two cell types inside an artificial tubular scaffold (Huang and Niklason, 2014); the shear forces associated with fluids flowing through the bioreactor cause the cells to assume shapes and features similar to those seen in real blood vessels (Traub and Berk, 1998). This technology has been widely used in cardiovascular research over the past decade (Piola et al., 2017; Punchard et al., 2009).

Robert et al. first bioengineered a 3D vessel from human muscle- and endothelial stem cells. Then they placed amyloid on the brain side of this bipartite model to measure how much of it moved to the blood side and also how much accumulated in the blood vessel. The results confirmed that ApoE2 helped to remove amyloid – in particular amyloid beta 42, which is the amyloid most associated with Alzheimer’s (McGowan et al., 2005) – much more efficiently than ApoE4. Moreover, Robert et al. discovered that adding high-density lipoprotein (HDL or 'good' cholesterol) helped to clear the amyloid, but only when ApoE was present.

The blood vessels in the bipartite model they built were more similar to those found in the body rather than those found in the brain, so they added other cells called astrocytes to make a tripartite model (Figure 1). In addition to removing toxins, astrocytes also have 'end feet' that wrap around blood vessels and become part of the blood-brain barrier. Intriguingly, the astrocytes in the tripartite model induced the endothelial cells to express cell markers and adopt a morphology similar to that observed in real cerebral blood vessels. The astrocytes also expressed human ApoE3 and, again, high-density lipoproteins helped these apolipoproteins to clear the amyloid.

**Figure 1.**
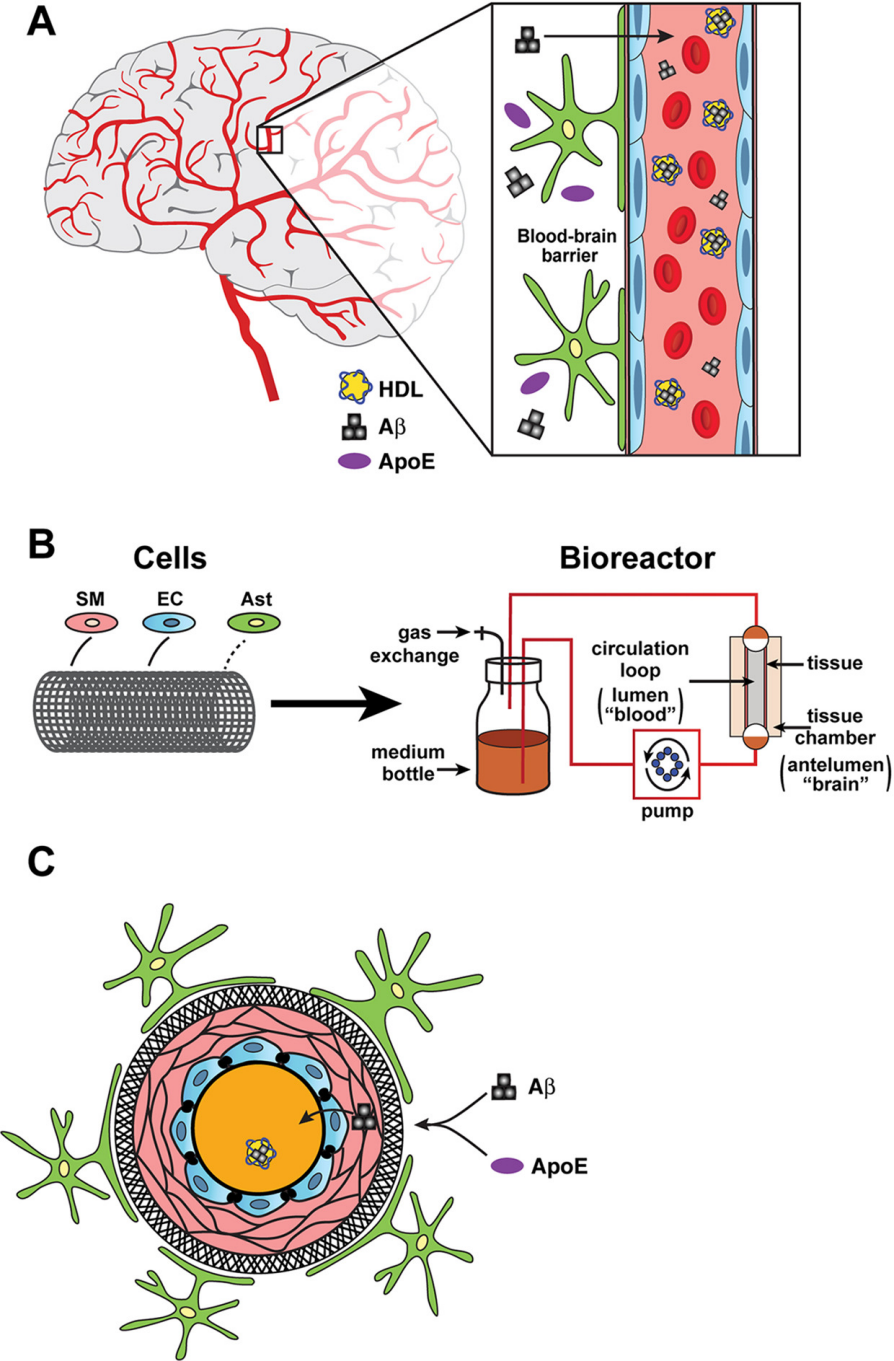
Bioengineering a model of the blood-brain barrier. (**A**) The blood-brain barrier separates the brain (grey or white background) from the the blood (pink background) inside the blood vessels in the brain. The main components of the barrier are smooth muscle cells (SM; narrow pink layers), endothelial cells (EC; blue), and astrocytes (green/yellow). The different isoforms of apolipoprotein E (ApoE; purple ovals) help to move beta-amyloid peptides (Aß; black) from the brain to the blood with different levels of efficiency, and with the help of high-density lipoproteins (HDL; yellow). (**B**) In the bioreactor, smooth muscle cells, endothelial cells, and astrocytes are grown on an artificial tubular scaffold (left) to mimic blood vessels. Fluid can then be pumped through the resulting bioengineered structure to mimic in vivo conditions and explore a range of cardiovascular phenomena. (**C**) Cross-sectional view of the bioengineered blood vessel. Smooth muscle cells and endothelial cells grow inside the scaffold (circular black structure), while astrocytes are grown on the outside of the scaffold. Panel A is redrawn from https://www.cldinc.com/portfolio/blood-brain-barrier-illustration/.

A powerful aspect of this technology is its ability to incorporate human tissues. One of the main struggles in Alzheimer’s research is the repeated failure of treatments developed in mouse models to work in human clinical trials. One big difference between mice and humans is their lipoprotein physiology: mice mainly have high-density lipoprotein (good cholesterol), whereas humans mostly have low-density lipoprotein (bad cholesterol; Yin et al., 2012). Robert et al.'s bioreactor allows human cells to be tested with human lipoproteins.

An immediate challenge is to explore the details of ApoE physiology: how, for example, do the different ApoE isoforms affect amyloid transport, and what components of the lipoprotein receptor signaling pathway are involved? However, the technology developed by Robert et al. could have broader applications. For example, it could be used to develop and test drugs that increase the transport of toxins from the brain to the blood, to test the delivery of drugs from the blood to the central nervous system, and to study the effects of conditions such as high blood pressure or low or high blood sugar on the brain (Barnes and Yaffe, 2011).
